# Thermal Conductivity of Suspended Graphene at High Temperature Based on Raman Spectroscopy

**DOI:** 10.3390/nano15191520

**Published:** 2025-10-05

**Authors:** Junyi Wang, Zhiyu Guo, Zhilong Shang, Fang Luo

**Affiliations:** 1 College of Advanced Interdisciplinary Studies & Hunan Provincial Key Laboratory of Novel Nano-Optoelectronic Information Materials and Devices, National University of Defense Technology, Changsha 410073, China; wangjunyi@nudt.edu.cn (J.W.); guozhiyu@nudt.edu.cn (Z.G.); shangzhilong23@nudt.edu.cn (Z.S.); 2 Nanhu Laser Laboratory, National University of Defense Technology, Changsha 410073, China

**Keywords:** suspended graphene, Raman spectroscopy, thermal conductivity

## Abstract

With the development of technology, many fields have put forward higher requirements for the thermal conductivity of materials in high-temperature environments, for instance, in fields such as heat dissipation of electronic devices, high-temperature sensors, and thermal management. As a potential high-performance thermal management material, studying the thermal conductivity of graphene at high temperatures is of great significance for expanding its application range. In this study, high-quality suspended graphene was prepared through PDMS dry transfer, which can effectively avoid the binding and influence of the substrate. Based on the calculation model of the thermal conductivity of suspended graphene, the model was modified accordingly by measuring the attenuation coefficient of laser power. Combined with the temperature variation coefficient of suspended graphene measured experimentally and the influence of laser power on the Raman characteristic peak positions of graphene, the thermal conductance of suspended graphene with different layers under high-temperature conditions was calculated. It is conducive to a further in-depth understanding of the phonon scattering mechanism and heat conduction process of graphene at high temperatures.

## 1. Introduction

In recent years, the swift advancement of nanotechnology has led to major miniaturization and integration of electronic components, which in turn presents new challenges for effectively managing heat dissipation in chip-based devices [1,2,3,4]. Graphene, an innovative two-dimensional material with atomic-layer thickness, demonstrates great application potential in nano-optoelectronic applications due to its exceptional physical properties, such as extremely high carrier mobility and exceptional thermal conductivity. Consequently, graphene is widely regarded as an ideal candidate for thermal management materials [5,6,7,8]. Suspended graphene structures can effectively avoid carrier scattering and phonon leakage induced by substrates, thereby preserving the material’s superior mechanical, electrical, and thermal properties.

The defects and disorder of graphene structure brought by traditional preparation methods may reduce its thermal conductivity [9]. The average thermal conductivity of corrugated graphene is about 27% lower than that of flat graphene [10,11]. Moreover, the presence of the substrate will suppress the thermal conductivity of graphene [12,13]. As the temperature rises, the thermal conductivity of graphene fluctuates within a certain temperature range, but generally shows a downward trend [14].

In 2008, Balandin et al. [15] measured the thermal conductivity of suspended monolayer graphene at room temperature. The results indicated that its thermal conductivity was much higher than that of diamond and graphite bulk materials, reaching 4840–5300 W·m^−1^·K^−1^. However, subsequent studies found that this experiment may have overestimated the Raman laser absorption power of graphene, resulting in a result that is 4–6 times larger. In 2010, Cai et al. [10] discovered that the thermal conductivity of suspended monolayer graphene ranged approximately from 2500 to 3100 W·m^−1^·K^−1^ at 350 K and from 1200 to 1400 W·m^−1^·K^−1^ at 500 K. The preparation methods of graphene lead to certain differences in its quality, which introduce additional phonon scattering and consequently affect thermal conductivity [16,17]. Graphene mechanically exfoliated typically exhibits higher thermal conductivity. Balandin [15] obtained monolayer graphene from mechanically exfoliated highly oriented pyrolytic graphite (HOPG) and suspended it. Its thermal conductivity was measured by Raman spectroscopy as 5300 ± 480 W·m^−1^·K^−1^, which is the highest thermal conductivity of known materials. Early studies suggested that thermal transport in monolayer graphene was almost entirely governed by LA/TA phonon modes [18,19]. However, subsequent research suggests that at low temperatures (T < 140 K), the thermal conductivity follows a distinct power-law dependence on temperature (κ ∝ T^1.53±0.18^) [20]. It indicates that in suspended monolayer graphene, heat transport is primarily dominated by ZA phonon modes under cryogenic conditions. Furthermore, at these temperatures, the thermal conductivity of graphene is not an intrinsic material property but exhibits a linear increase with sample length, highlighting the critical role of boundary scattering in low-dimensional systems.

Early theoretical calculations primarily relied on three-phonon scattering models. However, recent theoretical studies have revealed that even at room temperature, a large number of low-energy ZA mode phonons in monolayer graphene lead to its four-phonon scattering process, which cannot be ignored [21]. Feng et al. [22] found that the thermal conductivity of monolayer graphene at room temperature is only about 810 W·m^−1^·K^−1^ by introducing four-phonon scattering, which is much lower than the calculation result including only three-phonon scattering (about 3383 W·m^−1^·K^−1^).

At present, Raman spectroscopy and electron beam self-heating methods are the main methods in graphene thermal conductivity measurement. Raman spectroscopy is based on the absorption of Raman laser light by graphene, and the conductivity is calculated by measuring the change in Raman spectrum; while the estimation accuracy of laser absorption power of this method is high, there may be some problems, such as contact thermal resistance. The research group led by Xiangfan Xu at Tongji University measured the room-temperature thermal conductivity of multilayer molybdenum disulfide by the electron beam self-heating method [23]. This method avoids contact thermal resistance but is limited to room-temperature measurements and is sensitive to surface impurities.

Non-equilibrium phonon transport will make the measured thermal conductivity of graphene lower. Li Shi et al. and Xiulin Ruan et al. found that phonons of different modes are in a non-equilibrium state, which leads to a smaller thermal conductivity of graphene in actual measurements [24,25]. Bao Hua et al. calculated that when non-equilibrium phonon transport in graphene is ignored, the thermal conductivity of suspended graphene measured based on laser irradiation will be underestimated by 1.4–2.6 times [26].

The presence of a substrate can affect the thermal conductivity of graphene. Ruiling Zhang and Lin Gan et al. [27] measured the in-plane thermal conductivity of monolayer graphene on a silicon oxide substrate (~600 W·m^−1^·K^−1^ at 300 K). When graphene is supported on a substrate, the ZA phonon mode is suppressed, leading to a lower in-plane thermal conductivity compared to suspended graphene. Moreover, the influence of different substrates on the thermal conductivity of graphene varies significantly. Ge Daohan et al. [28] reported the in-plane thermal conductivity of multilayer graphene on a SiN substrate, ranging from 150 to 1250 W·m^−1^·K^−1^ at room temperature. Physicists David Goldhaber-Gordon from Stanford University, Wang Feng from the University of California, Berkeley, and Zhang Yuanbo from Fudan University, among others [16], have discovered signs of superconductivity in more readily available three-layer graphene sheets. Its apparent superconductivity is similar to that of traditional high-temperature superconductor copper-based materials. Lee et al. [29] found that as the grain size decreased, phonon scattering became severe, resulting in a reduction in the suspended thermal conductivity of graphene. Not only that, phonon scattering is also related to the grain boundary Angle *θ*. Chen et al. [30] deduced that the relationship between the specular reflection coefficient P and the interface Angle *θ* is *P* ≈ exp(−*C*1·sin2*θ*), and pointed out that in the case of small angles, as the grain boundary Angle increases, the specular reflection coefficient decreases and phonon scattering intensifies. The presence of impurities and defects in graphene can also induce modifications in its thermal transport properties. Li Baowen’s group deposited gold atoms on graphene and varied the coverage, observing that thermal conductivity initially decreased and then increased [31]. While at higher coverages, they form a conductive network, increasing thermal conductivity. Malekpour et al. [32] and Lee et al. [33] created uniform pores on graphene surfaces via electron beam irradiation, showing that thermal conductivity decreased with increasing pore area.

In 2011, Duhee et al. [17] from South Korea conducted a study on the thermal conductivity differences in suspended graphene with different pore sizes and different points of the same pore in substrate etching. Duhee et al. tested the thermal conductivity of suspended graphene in holes with diameters of 2.6, 3.6, 4.6 and 6.6 μm within the temperature range of 300 K to 500 K. The hole depth was 1.7 μm, which was sufficient to prevent laser interference reflected and scattered from the bottom of the hole. Research has found that as the aperture increases, its temperature decreases accordingly, resulting in a slower rise in the hole temperature and a gradual reduction in the frequency shift in characteristic peaks with the variation in laser power. Duhee et al. found that when the absorption rate was 2.3%, the thermal conductivity of monolayer suspended graphene at 350 K was approximately 1800 W·m^−1^·K^−1^, and its thermal conductivity at 500 K was approximately 710 W·m^−1^·K^−1^. When the absorption rate is 3.4%, the thermal conductivity of single-layer suspended graphene at 325 K is approximately 2700 W·m^−1^·K^−1^, and its thermal conductivity at 500 K is approximately 630 W·m^−1^·K^−1^.

Scholars have conducted extensive theoretical and experimental research on the thermal conductivity of graphene at different temperatures. However, there are still certain challenges in preparing high-quality suspended graphene and obtaining more accurate thermal conductivity data of suspended graphene. In this study, high-quality suspended graphene was prepared using the polydimethylsiloxane (PDMS) dry transfer method, avoiding substrate-induced carrier scattering and phonon leakage. Raman spectroscopy was employed to investigate the thermal conductivity of suspended graphene under varying temperatures and laser powers, providing foundational experimental data and theoretical insights into phonon transport, scattering mechanisms, and their temperature dependence.

## 2. Fabrication and Characterization of Suspended Graphene

**Figure 1:**
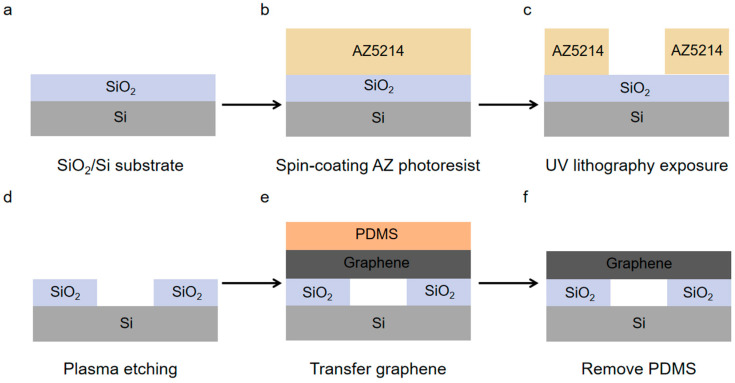
Schematic of dry-transfer fabrication for suspended graphene using PDMS. (**a**) SiO_2_/Si substrate. (**b**) Spin-coating of AZ photoresist. (**c**) Photolithographic patterning of AZ photoresist. (**d**) Etching of SiO_2_ layer to create a perforated substrate. (**e**) Dry transfer of exfoliated graphene with PDMS. (**f**) Removal of Thermal release for PDMS.

Figure 1 illustrates the PDMS dry transfer process of suspended graphene. The substrate’s pre-etched pore patterns were meticulously designed and arranged, with an etching depth of 0.5 μm and pore diameters ranging from 3 to 10 μm. These pores were organized into four distinct array elements to fabricate the photomask, which featured a downward-facing chromium film, with the graphic area remaining transparent and chromium-free. A 1 μm-thick silicon oxide wafer was employed as the substrate. Spin-coating AZ5214 photoresist on the surface of the silicon wafer, baking the surface at high temperature, using ultraviolet lithography for exposure, developing and fixing, and obtaining the required hole pattern. After that, the SiO_2_ layer is etched by inductively coupled plasma etching machine (ICP) to obtain porous substrates with different sizes. Finally, mechanically exfoliated highly oriented pyrolytic graphite (HOPG) flakes were peeled off to PDMS, which was subsequently aligned and adhered to the perforated substrate. Heating was applied to release the PDMS, thereby completing the fabrication of suspended graphene.

Structural characterization. The suspended graphene was characterized using both optical microscopy (OM) and scanning electron microscopy (SEM) to determine the pore dimensions and inspect the surface for potential defects. Subsequently, confocal Raman spectroscopy was performed under a chamber pressure of approximately 4.2 × 10^−4^ Pa. A 532 nm laser with an excitation power of 15 mW was employed, and the spectra were acquired with 10 accumulations at an integration time of 1 s per scan. The Raman measurements were conducted to determine the layer number of the suspended graphene.

High-temperature thermal conductivity measurement of suspended graphene. The Raman spectra of suspended graphene were measured under a fixed laser power of 15 mW while varying the temperature from 324 K to 824 K, and Raman spectra of suspended graphene were measured at 10 mW to 42 mW with a fixed ambient temperature of 824 K. the first-order temperature coefficients of the G peak and 2D peak for graphene were, respectively, fitted with their first-order variable power coefficient, and the thermal conductivity of graphene at different temperatures was further calculated, with the first-order power coefficient of G peak and 2D peak for graphene.

## 3. Results and Discussion

**Figure 2:**
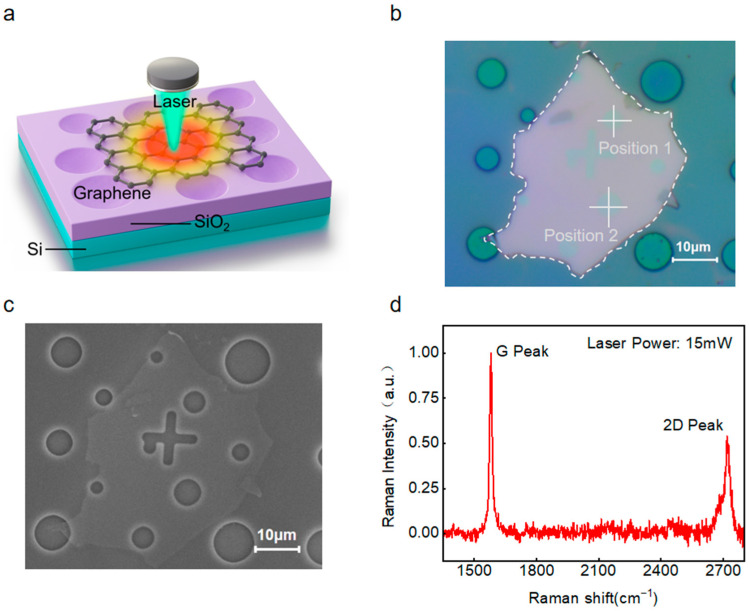
Structural characterization of suspended graphene. (**a**) Principle of Raman spectroscopy measurement. (**b**) Optical microscopy image of the suspended graphene. (**c**) Scanning electron microscopy (SEM) image of the suspended graphene. (**d**) Raman spectrum of the sample spanning the cavity region.

We placed the fabricated suspended graphene inside a vacuum chamber and measured its Raman spectra using a 532 nm laser, as shown in Figure 2a. Figure 2b provides an optical microscopy image of the suspended graphene, revealing a uniform thickness across areas where it was transferred over holes of different sizes. For our analysis, we focused on the suspended section over a 4.5 μm hole. The SEM image in Figure 2c confirms that this part of the sample is flat and free of defects, with a smooth surface over the opening. We used confocal Raman spectroscopy to examine the suspended region, and the resulting scope is shown in Figure 2d. This scope displayed characteristic peaks with an intensity ratio I_2D_/I_G_ of 0.312 and a 2D peak full width at half maximum (FWHM) of 56.7 cm^−1^. Based on these spectral features and established criteria, we identified the sample as four-layer graphene.

**Figure 3:**
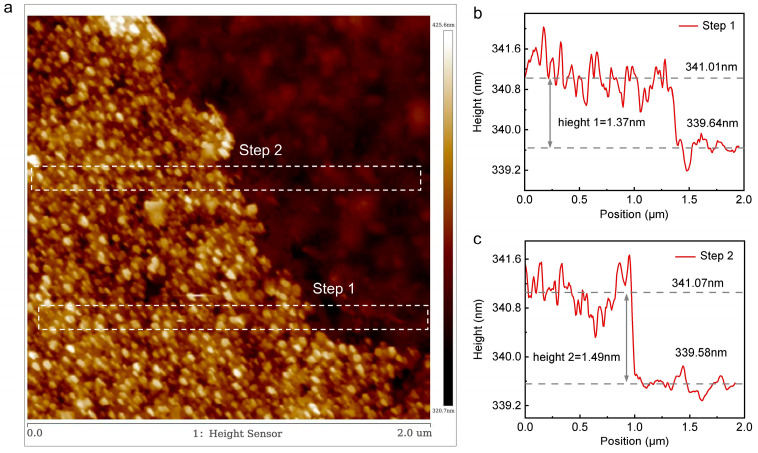
Atomic force microscopy (AFM)images of the edges of the suspended graphene samples. (**a**) The surface morphology of the graphene sample. (**b**) Step height at position 1. (**c**) Step height at position 2.

At room temperature, we characterized the morphology of the sample using the dynamic mode (i.e., tapping mode) of a Bruker atomic force microscope. As shown in Figure 3, we measured the step height between the sample edge and the substrate in two different positions and obtained an average value of approximately 1.43 nm, which is slightly larger than the theoretical value of 1.34 nm. The AFM topography image of the sample surface reveals that there are some tiny particle impurities on the SiO_2_/Si substrate. These tiny particle impurities, attached to both the bottom and top surfaces of the graphene, cause the measured sample thickness to be slightly larger than the theoretical value. Based on the AFM images and Raman spectroscopy information of the sample, we have determined that the sample is four-layer suspended graphene.

**Figure 4:**
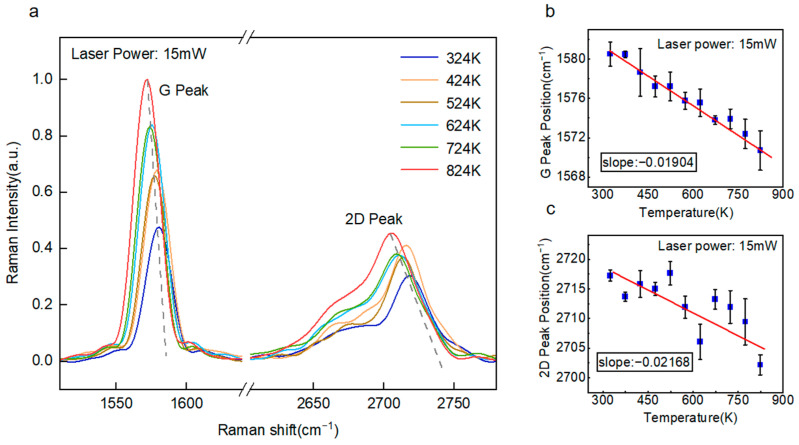
Calibration of first-order temperature coefficient for suspended graphene samples. (**a**) Raman spectra of suspended graphene across the temperature range of 324–824 K. (**b**) First-order temperature coefficient of the G peak in suspended graphene. (**c**) First-order temperature coefficient of the 2D peak position in suspended graphene.

Following structural characterization, the first-order temperature coefficient *χ*1 and power-dependent coefficient ∂*ω*_1_/∂*P* of the four-layer suspended graphene were calibrated. With the laser power fixed at 15 mW, single-point Raman measurements were conducted at varying temperatures. Figure 4a presents the Raman spectra of the suspended sample across a temperature range of 324 K to 824 K. As the ambient temperature increased, the full width at half maximum FWHM of the 2D peak broadened, while both the G and 2D peaks exhibited significant red shifts. When quantitatively calculating the frequency shift in Raman characteristic peaks, some characteristic peaks in the Raman spectrum show a certain degree of asymmetry. Considering that the Raman peaks of crystal materials are often affected by crystal structure and phonon mode, their peak shapes are closer to the Lorentz distribution. Therefore, in this study, we adopted the Lorentz type fitting.

As illustrated in Figure 4b,c, the Raman peak positions of the suspended graphene demonstrated a linear dependence on temperature. Linear fitting yielded the first-order temperature coefficient for 2D Peak and G Peak: *χ*_12*D*_ = 0.02168 cm^−1^·K^−1^, *χ*_1*G*_ = 0.01904 cm^−1^·K^−1^. The G peak corresponds to the in-plane carbon-carbon double bond (C=C) stretching mode, whereas the 2D peak arises from a double-resonance phonon process. Consequently, the 2D peak exhibits greater sensitivity to temperature variations, as evidenced by its larger shift magnitude. However, in few-layer graphene, the symmetry of the 2D peak gradually diminishes with increasing layer number. Thus, for temperature monitoring in few-layer systems, the G peak may serve as a more reliable indicator.

**Figure 5:**
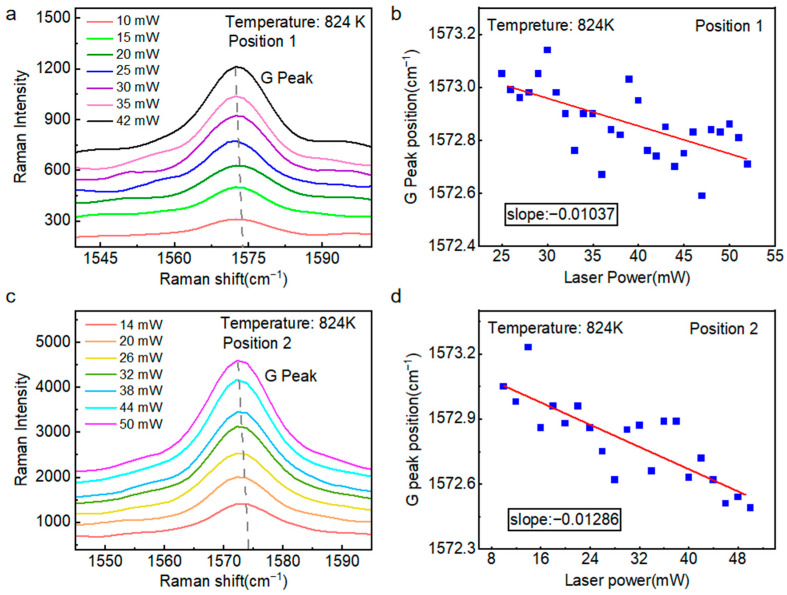
First-order power coefficient at positions 1 and 2 (with different hole diameters) of four-layer suspended graphene (Sample 1). (**a**) Raman G peak of suspended graphene position 1 at a temperature of 824 K under various laser power densities. (**b**) Shift in the G peak position of suspended graphene position 1 as a function of increasing laser power density at 824 K. (**c**) Raman G peak of suspended graphene position 2 at a temperature of 824 K under various laser power densities. (**d**) Shift in the G peak position of suspended graphene position 2 as a function of increasing laser power density at 824 K.

With the temperature fixed at 824 K, we performed Raman measurements on the suspended graphene under varying laser power. Figure 5a presents the G peak position in Raman spectra acquired at laser powers ranging from 10 to 42 mW. The increasing laser power induced localized heating in the suspended graphene, resulting in lattice thermal expansion. This lattice distortion led to a systematic decrease in vibrational frequency, manifesting as a pronounced red-shift in the characteristic Raman peaks, as depicted in Figure 5b. Quantitative analysis yielded a first-order power coefficient of ∂*ω*_1*a-G*_/∂*P* = −0.01037 cm^−1^·mW^−1^. As shown in Figure 5c, we continue to perform Raman spectroscopy on sample 1 at position 2, and the first-order power coefficient ∂*ω*_1*b-G*_/∂*P* = −0.01286 cm^−1^·mW^−1^.

From the Raman spectrum, no defect peak D peak appears. The possible reasons are as follows in three aspects: Firstly, in this study, suspended graphene was prepared through the dry transfer of polydimethylsiloxane (PDMS), effectively avoiding the contamination of suspended graphene and the breakage of graphene caused by doping, surface tension, etc. It also prevented chemical corrosion and mechanical damage during the transfer process. Compared with traditional methods, the quality of the prepared graphene films is higher.

Secondly, in this study, when conducting Raman spectroscopy tests, suspended graphene was placed in a vacuum chamber. The presence of calcium fluoride glass in the vacuum chamber would lead to the attenuation of laser energy, which has not caused obvious damage to the suspended graphene sample.

Thirdly, the temperature inside the vacuum chamber is too high, resulting in significant thermal noise in the Raman spectrum of suspended graphene measured. Coupled with the fact that the intensity of the original D peak is too low, it is impossible to distinguish the original shape of the D peak.

**Figure 6:**
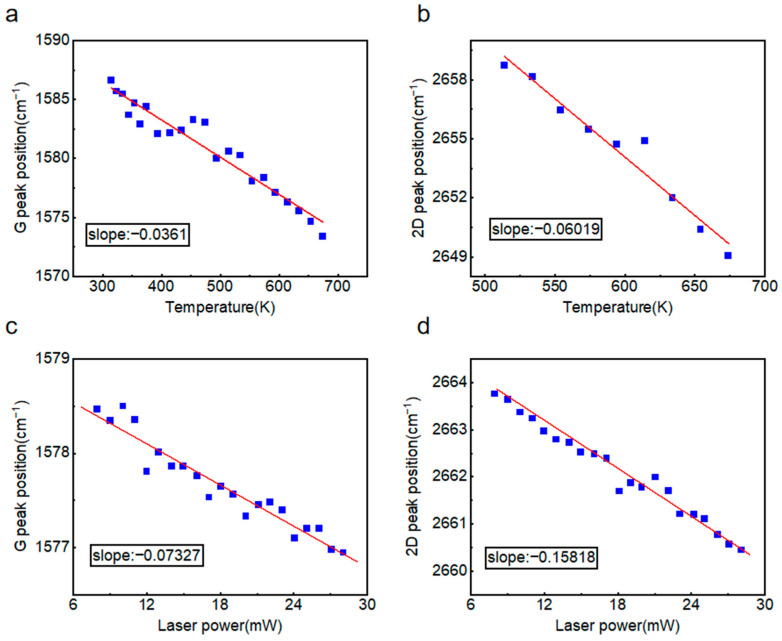
First-order temperature coefficient and power coefficient of monolayer suspended graphene. (**a**) Temperature-dependent shift in the graphene G peak. (**b**) Temperature-dependent shift in the graphene 2D peak. (**c**) Laser power-dependent shift in the graphene G peak. (**d**) Laser power-dependent shift in the graphene 2D peak.

Temperature-dependent Raman measurements were also conducted on monolayer suspended graphene under a constant laser power of 15 mW. As shown in Figure 6a,b, the first-order temperature coefficient for the G peak and 2D peak were determined to be *χ*_2-*G*_ = −0.0361 cm^−1^·K^−1^ and *χ*_2-2*D*_ = −0.06019 cm^−1^·K^−1^, respectively. Consistent with observations in four-layer suspended graphene, the 2D peak demonstrated greater temperature sensitivity than G peak. Power-dependent Raman spectroscopy was subsequently performed at 474 K, ∂*ω*_2-*G*_/∂*P* = −0.07327 cm^−1^·mW^−1^ and ∂*ω*_2-2*D*_/∂*P* = −0.15818 cm^−1^·mW^−1^, as shown in Figure 6c,d. Given the substantial measurement uncertainties associated with the 2D peak position, which is highly sensitive to factors such as carrier concentration and strain, the G peak data were exclusively employed for the calculation of thermal conductivity to ensure reliability.

The temperature rise measured in suspended graphene using a laser beam is attributed to the optical absorption by the supporting substrate. For substrate-supported graphene, the substrate interaction can reduce the mean free path of phonons in graphene, particularly for long-wavelength phonons, generally rendering it smaller than the laser spot size. Based on this, Cai Weiwei et al. [34], following the fundamental principles of optothermal Raman spectroscopy, assumed diffusive phonon transport in the suspended region while neglecting thermal losses due to surrounding air and radiation from graphene. They derived the temperature distribution in suspended graphene using finite difference methods and established the corresponding thermal diffusion equation:

(1) $$T\left(r\right)=T_{1}+\frac{Q}{2\pi \kappa D}\ln\left(\frac{R}{r}\right)\beta \left(r\right),r\leq R$$

(2) $$\kappa =\frac{ln(\frac{R}{r_{0}})}{2\pi DR_{g}}\alpha$$

In Equations (1) and (2), *T*(*r*) represents the temperature distribution of the suspended graphene in the holes, *T*_1_ represents the temperature at the boundary of the suspended graphene, *β*(*r*) is the spatial distribution function of the laser intensity. *R* denotes the radius of the etched hole, *r*_0_ represents the laser spot radius, *D* corresponds to the thickness of graphene, *R_g_* is the equivalent thermal resistance of graphene, and *α* is a coefficient determined by Equation (3):

(3) $$\alpha =\frac{T_{m}-T_{1}}{T_{0}-T_{1}}\beta (r_{0})$$

In Equation (3), *T*_0_ denotes the temperature at the edge of the laser spot and *T_m_* corresponds to the temperature at the center of the suspended graphene region. The calculation of *β*(*r*_0_) is given by Equation (4):

(4) $$\beta \left(r_{0}\right)=1+\frac{Ei\left(-1\right)-Ei(-\frac{R^{2}}{r_{0}^{2}})}{2ln(\frac{R}{r})}$$

*E_i_*(*x*) denotes the exponential integral. Since the temperature at the center of the suspended graphene *T_m_* cannot be directly substituted into Equation (1) for calculation, we derive Equations (5) and (6) by incorporating the governing equation with the Gaussian temperature distribution formula:

(5) $$T(m)\approx \frac{\int_{0}^{R}T(r)exp(-\frac{r^{2}}{r_{0}^{2}})rdr}{\int_{0}^{R}exp(-\frac{r^{2}}{r_{0}^{2}})rdr}$$

(6) $$\frac{T_{m}-T_{1}}{T_{0}-T_{1}}=\frac{\int_{0}^{R}ln(\frac{R}{r})\beta (r)re^{-\frac{r^{2}}{r_{0}^{2}}}dr}{ln(\frac{R}{r_{0}})\beta (r_{0})\int_{0}^{R}re^{-\frac{r^{2}}{r_{0}^{2}}}dr}$$

The formula for the calculation of the laser beam radius is

(7) $$r_{0}=\frac{0.61\lambda }{NA}$$

In our study, we used an Olympus 50× objective lens with a numerical aperture NA of 0.5. Using 532 nm green laser illumination with a numerical aperture (NA) of 0.5, the laser spot radius *r*_0_ is determined to be approximately 649 nm.

The thermal resistance *R_g_* is defined as the ratio of the temperature difference between two ends of the specimen to the applied heating power. Based on the temperature difference equation (Equation (8)), the equivalent thermal resistance *R_g_* is calculated as expressed in Equation (9):

(8) $$T_{m}-T_{1}=\frac{\partial T}{\partial \omega }\frac{\partial \omega }{\partial Q_{absord}}Q_{absord}=\frac{\partial T}{\partial \omega }\frac{\partial \omega }{\partial P_{absord}}P_{absord}=\frac{1}{\chi }\frac{\partial \omega }{\partial P_{absord}}P_{absord}$$

(9) $$R_{g}=\frac{T_{m}-T_{1}}{P_{absord}}=\frac{\partial T}{\partial \omega }\frac{\partial \omega }{\partial P_{absord}}=\frac{1}{\chi }\frac{\partial \omega }{\partial P_{absord}}$$

*χ* is the first-order temperature coefficient obtained by fitting experimental data. In addition, *P_absord_* is the heat source power absorbed by graphene, and there is an attenuation coefficient *a* between *P_absord_* and the laser power set at the time of measurement. Therefore, the actual first-order power coefficient of suspended graphene ∂*ω/∂P_absord_* = (∂*ω*/∂*P*)·*a*, where ∂*ω*/∂*P* is the experimentally measured first-order power coefficient. The attenuation coefficient of laser can be divided into two parts, one is the ratio of the laser power set by the instrument to the laser power actually received by the focusing plane of the 50-fold objective lens—*a*_1_ and the other is the ratio of the laser power before the laser enters the vacuum heating cavity to the laser power focused on the sample through the vacuum cavity surface—*a*_2_.

**Figure 7:**
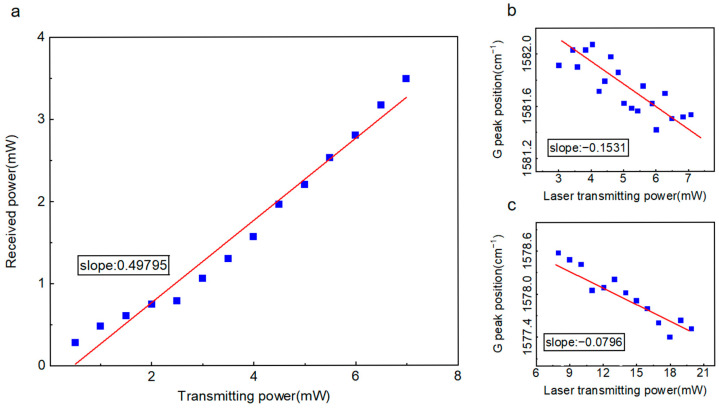
Calibration of laser power attenuation coefficient *a*. (**a**) Determination of attenuation coefficient *a*_1_. (**b**) Raman spectral measurement of ∂*ω*/∂*P* for suspended graphene in ambient atmosphere. (**c**) Raman spectral measurement of ∂*ω*/∂*P* for suspended graphene in vacuum chamber.

The attenuation coefficient *a*_1_ was experimentally determined using an optical power meter. The power meter was positioned on the sample stage and carefully adjusted such that its detection surface coincided with the focal plane of the 50× objective lens. By recording the meter readings at various laser output powers, we obtained *a*_1_ = 0.49753, as shown in Figure 7a. This coefficient *a*_1_ quantitatively represents the combined effects of laser emission efficiency, along with the propagation loss, scattering, and reflection occurring during laser transmission to the focal plane.

For Raman spectroscopic measurements conducted within the vacuum chamber, the precise determination of the secondary attenuation coefficient *a*_2_—defined as the power ratio between incident laser power at the chamber entrance and transmitted power reaching the graphene sample—is complicated by scattering and reflection losses at the CaF_2_ optical window and chamber interfaces. Under ambient conditions, the Raman power coefficient reflects only the primary attenuation: (∂*ω*/∂*P_transmit_*)·*a*_1_, where *P_transmit_* represents the nominal laser power set by the instrument. The vacuum chamber measurements incorporate both attenuation components: (∂*ω*/∂*P_transmit_*)·*a*_1_·*a*_2_. Through comparative analysis of the ambient and vacuum measurements in Figure 7b,c, we establish the secondary attenuation coefficient without requiring direct measurement of the complex vacuum interface losses: *a*_2_ = 0.5199. The total attenuation coefficient is consequently determined as *a* = *a*_1_·*a*_2_ = 0.25867.

According to the first-order temperature coefficient and first-order power coefficient of single-layer and four-layer suspended graphene, thermal conductivity measurements were performed at temperatures of 474 K and 824 K. Respectively, we finally measured that the thermal conductivity of the single-layer suspended graphene at 474 K was 1799.34 W·m^−1^·K^−1^. The thermal conductivities of the four-layer suspended graphene at 824 K were measured to be 673.85 W·m^−1^·K^−1^ (Position 1, R = 2.25 μm) and 646.97 W·m^−1^·K^−1^ (Position 2, R = 3.25 μm).

Because the thermal conductivity calculation model used in the calculation ignores the thermal conduction of air, the actual temperature of the suspended graphene surface is slightly smaller, which is equivalent to the measured laser attenuation coefficient being smaller than the actual one, and finally, the measured thermal conductivity is smaller. At the same time, the size of suspended graphene samples will also affect the heat conduction of air to a certain extent, resulting in deviation between the measurement results and the actual situation.

Combining the research results of Duhee et al. [17] on the thermal conductivity of suspended graphene with different pore sizes at different temperatures in 2011 and conducting a comparative analysis with the results of this study, we found that as the aperture increases, when the laser power changes, the variation amplitudes of the 2D peak and G peak gradually decrease, resulting in a gradual increase in the thermal conductivity of the ultimately measured suspended graphene. Meanwhile, the first-order temperature coefficient *χ*_2*D*_ of the 2D peak is larger than that of the G peak, indicating that the 2D peak is more dependent on temperature. This point was also confirmed in the research of Duhee et al. In this study, the thermal conductivity of single-layer suspended graphene at around 500K differs significantly from the research results of Duhee et al. Since the 2D peak is more sensitive to temperature, Duhee et al. calibrated the first-order temperature coefficient *χ* by studying the relationship between the 2D peak and temperature.

Previous scholars also measured the thermal conductivity of graphene prepared by different methods through various means. They summarized the research results of previous scholars and compared and analyzed them with this study. The summary is shown in Table 1. The thermal conductivities of suspended graphene prepared by different methods vary [15,17,34,35]. Not only that, even if the same preparation method is adopted, different measurement methods will also lead to differences in the thermal conductivity of the suspended graphene measured [36,37].

## 4. Conclusions

In addition to addressing the challenges of complex fabrication, low yield and its structural instability in suspended graphene production, this work established an original PDMS dry-transfer technique to fabricate high-quality suspended graphene for thermal transport measurements by Raman spectroscopy. All measurements displayed linear relationships against laser power of the Raman peak positions, as well as red shifts and broadening of both G and 2D Raman peaks in Fig. The experimental results show the thermal conductivities of monolayer suspended graphene for 1799.34 W·m^−1^·K^−1^ at 474 K and four-layer supported graphene adhesive on the substrate for 624.79 W·m^−1^·K^−1^ at 824 K, which give fundamental experimental data and are necessary to understand phonon benchmarking and scattering nature properties of graphene as well as temperature-dependent thermal properties.

## Figures and Tables

**Table 1:** Comparative Analysis of the Thermal Conductivity of Graphene.

Preparation Methods	Testing Methods	*T*/K	Layers	Thermal Conductivity/W·m^−1^·K^−1^	Ref.
Mechanical Exfoliation	Raman Spectroscopy	824	1	~1800	This work
		474	4	~660	
	Raman Spectroscopy	325	1	~1800	Duhee et al. [34]
		500	1	~710	
	Raman Spectroscopy	RT	1	~4840–5300	Balandin et al. [15]
	Suspended thermal bridge	RT	2	~600	Pettes Michael Thompson et al. [36]
CVD	Raman Spectroscopy	350	1	~2500	Cai et al. [34]
		500	1	~1400	
	Raman Spectroscopy	350	1	~2600–3100	Chen et al. [35]
Mechanical Exfoliation, CVD	Thermal Analytical Techniques	1000	1	~310	Dorgan Vincent E et al. [37]

## Data Availability

Data are contained within the article.
